# IgG4-Related Kidney Disease Associated With End-Stage Kidney Disease, Renal Pseudotumor, and Renal Vein Thrombosis

**DOI:** 10.7759/cureus.22837

**Published:** 2022-03-04

**Authors:** Mohammed Tawhari, Nourah Al Oudah, Yousof Al Zahrani, Mansoor Radwi

**Affiliations:** 1 Internal Medicine: Nephrology, King Saud Bin Abdulaziz University for Health Sciences, College of Medicine, Riyadh, SAU; 2 Research, King Abdullah International Medical Research Center, Riyadh, SAU; 3 Internal Medicine: Nephrology, King Abdulaziz Medical City, Riyadh, SAU; 4 Pathology, King Abdulaziz Medical City, Riyadh, SAU; 5 Radiology, Ministry of National Guard - Health Affairs, Riyadh, SAU; 6 Internal Medicine • Hematology, College of Medicine- University of Jeddah, Jeddah, SAU

**Keywords:** renal vein thrombosis, plasma cell tumor, end stage kidney disease (eskd), igg4 related pseudotumor, igg 4 disease

## Abstract

IgG4 related disease (IgG4-RD) is a systemic autoimmune disease characterized by tissue invasion with IgG4-producing plasma cells, resulting in tissue dysfunction. IgG4-RD can affect the kidney in various forms, including renal mass, tubulointerstitial disease, and glomerulonephritis. IgG4-RD can mimic other autoimmune diseases and neoplasms, and as such, maintaining a high index of suspicion is the key to timely diagnosis and treatment. In this paper, we present a case of IgG4-RD that presented with pseudotumor and severe renal dysfunction that progressed to end-stage kidney disease (ESKD), associated with a rare finding of renal vein thrombosis (RVT).

## Introduction

IgG4 related disease (IgG4-RD) is a multisystem autoimmune disorder characterized by tissue infiltration with IgG4 producing plasma cells, resulting in organ dysfunction [1-2]. IgG4 Related Kidney Disease (IgG4-RKD) manifestations vary, with acute tubulointerstitial nephritis being the commonest. It can also cause glomerular disease or retroperitoneal fibrosis resulting in ureteric obstruction and hydronephrosis [1-3]. Occasionally, IgG4-RKD can present as renal masses mimicking renal cell carcinoma [2,4].

Low-dose glucocorticoids are the first-line treatment for IgG4-RD, and timely diagnosis and therapy can avoid unnecessary surgery and preserve organ function. Most patients achieve partial or complete remission; however, up to 35% of patients relapse once steroids are tapered off and require further treatment with other agents such as Rituximab [2,4].

We describe a case of IgG4-RKD manifesting with renal dysfunction and a renal mass that progressed to End-Stage Kidney Disease. We also report the unique finding of left renal vein thrombosis (RVT), which to our knowledge, has never been described previously with IgG4-RKD.

## Case presentation

A 55-year-old lady presented with acute kidney injury (AKI). Her history was unremarkable, she was not on any medication, and she had no constitutional symptoms. Her Blood Pressure (BP) was elevated, 197/110, but the rest of her vital signs and physical were normal. The patient's laboratory investigations upon admission are shown in table 1.

**Table 1:** Patient's laboratory investigations at the time of admission. BUN: Blood Urea Nitrogen; CRP: C-Reactive Protein; ESR: Erythrocyte Sedimentation Rate; IgG: Serum Immunoglobulin G: UPCR: Urine protein/creatinine ratio: URBCs: Urine Red Blood Cells: ANA: Antinuclear Antibodies; Anti-dsDNA: Anti-double stranded DNA; HPF: High Power Field

Test	Patient value	Reference range
White Blood Count (WBC)	12.8 x10^9^/L	4-11 x10^9^/L
Hemoglobin (Hgb)	98 gm/L	120-160 gm/L
Platelet count	178 x10^9^/L	150-400 x10^9^/L
Serum Albumin	37 g/L	35-52 g/L
Bicarbonate	7 mmol/L	22-29 mmol/L
serum Creatinine	1041 umol/L	50-98 umol/L
Blood Urea Nitrogen (BUN)	36.3 mmol/L	3.5-7.2 mmol/L
C-Reactive Protein (CRP)	93 mg/L	< 8 mg/L
Erythrocyte Sedimentation Rate (ESR)	120 mm/hr	0-30 mm/hr
Serum Immunoglobulin G (IgG)	23.6 g/L	7.5-15.6 g/L
Urine protein/creatinine ratio (UPCR)	2.33	<0.2
Urine Red Blood Cells (URBCs)	7 cells/High Power Field (HPF)	0-5 cells/HPF
Antinuclear Antibodies (ANA)	249.03 units	< 20 units is negative 20-60 units is borderline > 60 is strongly positive
Anti-double stranded DNA (Anti-dsDNA)	717 International Unit/ml (IU/ml)	< 200 IU/ml is negative 201-300 IU/ml is borderline >300 IU/ml is positive

**Figure 1:**
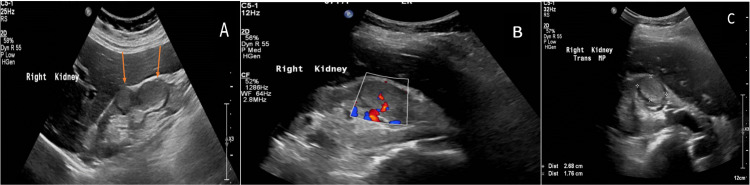
A, B, and C: Sagittal and transverse greyscale and Doppler ultrasound images of the right kidney showing two lobulated hyperechoic masses of the interlobar region and lower pole (arrows).

Abdominal ultrasound showed increased renal echogenicity with an atrophied left kidney. The right kidney measured 12 cm with two hypoechoic lesions, one at the interpolar area measuring 2.5x 2 cm and one at the lower pole measuring 2.5 x 1.6 cm (Figure 1).

**Figure 2:**
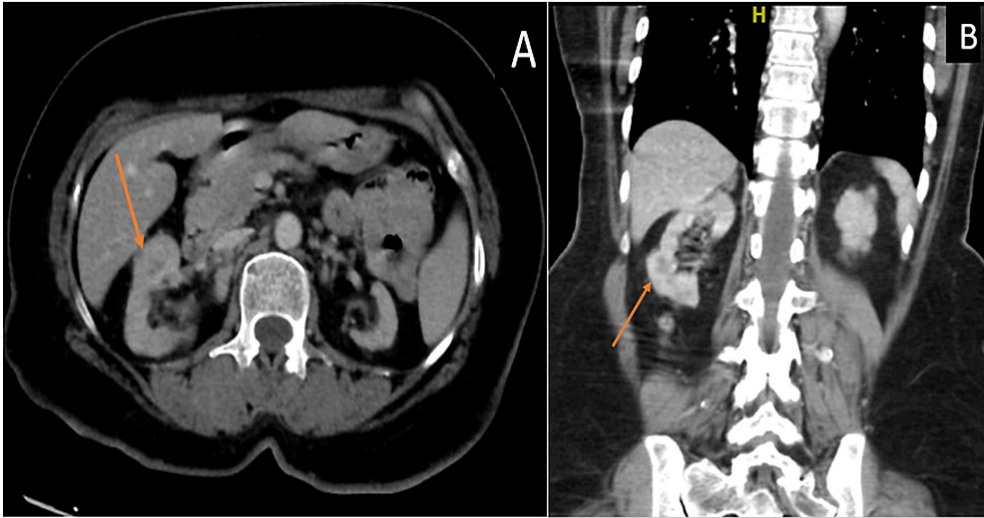
A & B: Contrast-enhanced axial and coronal CT scan images of the abdomen showing a heterogenous slightly hypodense mass of the right kidney (arrow).

Subsequently, a contrast-enhanced CT scan of the abdomen and pelvis showed focal thickening of the mid anterior right renal cortex and a heterogenous slightly hypodense 2 cm mass of the right kidney suspicious for papillary renal cell cancer or pseudotumor (Figure 2).

**Figure 3:**
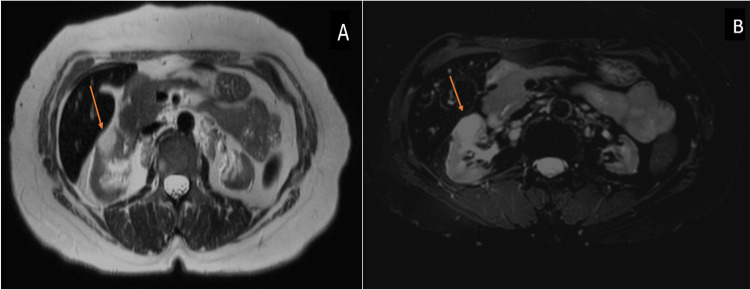
A & B: Axial T2 and T2 fat sat MRI images of the abdomen showing a hyperintense mass of the right kidney (arrow). The left kidney is atrophic.

There was an exophytic hypovascular renal mass in the left kidney with multiple enlarged para-aortic lymph nodes and a porta hepatis cystic lesion. Further evaluation by magnetic resonance imaging showed bilateral hyperintense renal lesions with cortical edema consistent with an inflammatory or infection process, e.g., tuberculosis (TB) or fungal infection (Figure 3).

**Figure 4:**
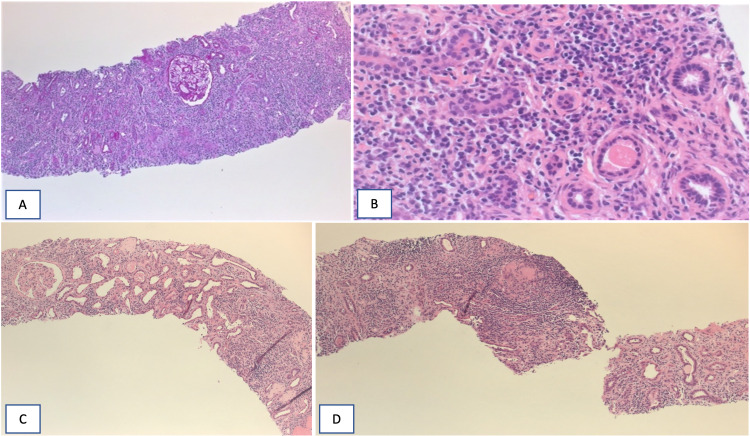
A, B, C& D: A. Low power (X10) show heavy interstitial infiltrate of lymphocytes and plasma cells associate with severe IF/TA. B. High power (x40) shows tense plasma cell infiltrate in background of interstitial scarring and tubular atrophy. C. Low power (x10) shows storiform fibrosis. D. Low power (x10) shows tubular granuloma.

Due to the uncertainty, a percutaneous needle biopsy was obtained. The biopsy showed severe interstitial lymphoplasmacytic infiltration with severe interstitial fibrosis and tubular atrophy (Figure 4A, 4B), storiform fibrosis (Figure 4C), and noncaseating granuloma formation (Figure 4D). The glomeruli were preserved. Immunohistochemical staining and flow cytometry in the tissue sample was negative for lymphoma and fungal, and TB cultures were negative.

**Figure 5:**
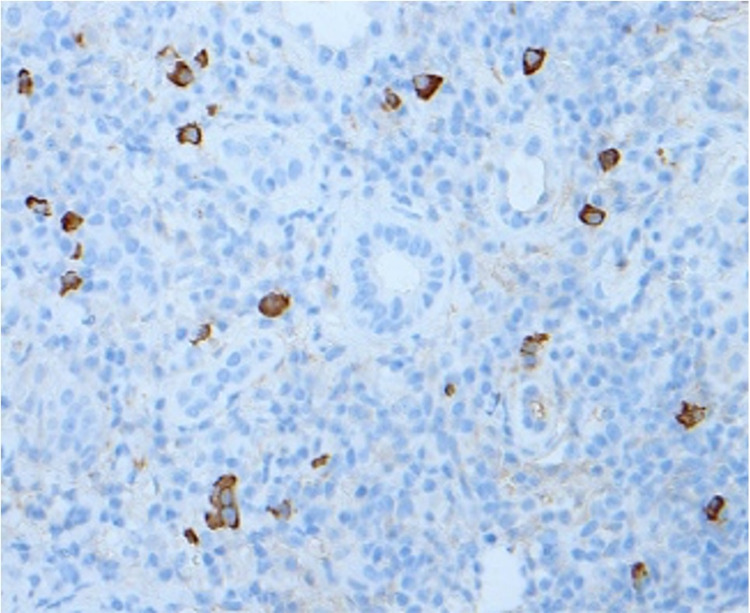
Immunohistochemistry showing >20 IgG4 positive plasma cells (EP138) /HPF.

Upon request, immunohistochemistry staining showed more than 20 IgG4-positive plasma cells per high power field (Figure 5).

**Figure 6:**
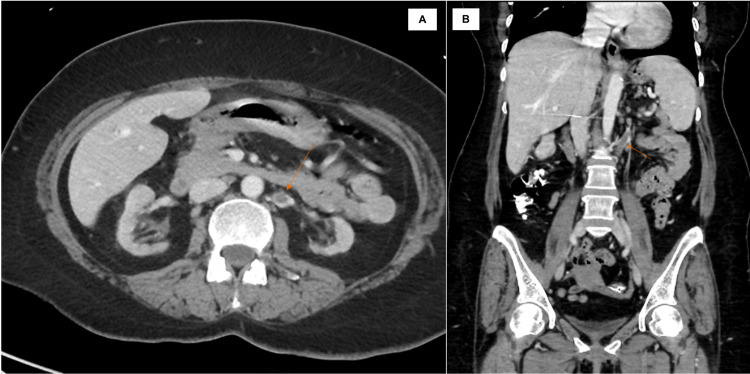
A &B: Axial and sagittal contrast-enhanced CT showing left renal vein Thrombosis (arrow) and resolution of the renal masses.

Due to severe renal impairment, hemodialysis was initiated along with prednisone 0.6 mg/kg. Unfortunately, she remained dialysis-dependent after one month of steroid therapy. Nevertheless, the renal mass, lymphadenopathy, and portahepatis cyst resolved in follow-up CT (Figure 6). Interestingly, the follow-up CT showed left RVT (Figure 6). Steroids were gradually tapered over three months, and the patient progressed to ESKD.

## Discussion

In this paper, we report a case of IgG4-RKD with pseudotumor and RVT that progressed to ESKD. To our knowledge, only 3 cases have been reported in Saudi Arabia [5-7]. We believe that this case report is interesting for the following reasons: 1) IgG4-RKD typically affects older men, but in this case, the patient is a woman, 2) IgG4-RKD led to ESKD 3) this is the first report of the association of IgG4-RKD and RVT, and 4) high index of suspicion allowed for accurate diagnosis and avoided unnecessary nephrectomy.

IgG4-RD typically affects middle-aged to older-age individuals, with male predominance [3]. Maintaining a high index of suspicion is the key to a timely diagnosis. Diagnosing IgG4-RKD is challenging and can be confused with other systemic lymphonodular disorders and autoimmune diseases [8]. Kawano et al. proposed the following criteria to diagnose IGG4-RKD: a) organ dysfunction/damage b) elevated total IgG or IgG4 subclass c) radiological findings including multiple hypodensities, hypovascular mass or renal enlargement and d) histopathological: IgG4 positive plasma cells greater than 10 per high power filed or the ratio of IgG4 positive to IgG4 negative plasma cells greater than 40%, and storiform fibrosis [9]. Immunohistochemistry examination is highly sensitive and specific (over 90% sensitive and specific) [10]. Our patient met all the proposed criteria, the flowcytometry ruled out lymphoproliferative disorders, and serology excluded vasculitis. The presence of storiform fibrosis, which is found in up to 92% of the cases of IgG4-RKD, further strengthens our diagnosis of IgG4 disease [8]. 

Although our patient had strongly positive ANA and Anti-DNA serology, the absence of extra-renal manifestation of lupus, normal complements level, and glomeruli spares make lupus nephritis unlikely. However, the lupus markers would need to be followed up periodically.

Previous reports have shown that renal involvement in IgG4-RKD is commonly accompanied by other organ involvement [2]. A different organ can be affected synchronous or meta-synchronous [10]. In this case, there was the involvement of lymph nodes and the porta hepatis. The resolution of the lymphadenopathy and the cystic lesions with steroids support the manifestation of IgG4-RD.

The IgG4-RD has been thought to follow a relatively benign course; however, some patients can develop a poor outcome. In one case series of 35 patients with IgG4-RKD, two patients required hemodialysis and progressed to ESKD, despite corticosteroid therapy [10]. Interestingly, these two patients had higher serum creatinine. Hence, severe kidney impairment can predict an unfavorable outcome. Also, patients with extensive tubulointerstitial nephritis, particularly if they have small kidneys, are less likely to respond to steroids [1]. Our patient had markedly elevated serum creatinine and atrophied left kidney, which may explain the poor response to therapy. 

We believe that the lack of response to steroids was due late presentation of severe IgG4 disease, as evidenced by the presence of extensive tubulointerstitial infiltration and severe interstitial fibrosis. The diagnosis of IgG4-RKD that manifests with a renal mass is often made after nephrectomy [4,11]. Maintaining a high index of suspicion, in this case, avoided unnecessary nephrectomy and preserved residual renal function [4,12]. Resolution of IgG4 related mass with treatment can vary between 1-12 months [3]. In this case, the renal masses and other masses resolved in one month of steroid treatment. 

Lastly, our patient developed left RVT, possibly as a complication of IgG4-RKD. Thromboembolic events in the setting of IgG4-RD have been reported [13]. However, RVT, particularly in the absence of nephrotic syndrome, has never been reported. Of note, the antiphospholipid antibody testing was negative, so the diagnosis of antiphospholipid antibody syndrome was unlikely. There are a few possible explanations. The mass effect causes pressure on the adjacent blood vessels leading to venous stasis, the involvement of the blood vessels by the IgG4 inflammatory process leading to endothelial injury and thrombosis, and the possibility of a separate hypercoagulable process. We are not convinced that the RVT explained the progression to ESKD, as the thrombosis involved the left kidney, which was atrophied and likely poorly functioning.

## Conclusions

IgG4-RKD should be considered in any renal lesion differential diagnosis, mainly if it exhibits atypical features of malignancies, regardless of age, gender, or ethnicity. Maintaining a high index of suspicion is the key to timely diagnosis and treatment, which can preserve organ function and avoid unnecessary surgery. IgG4-RKD can be associated with renal vein thrombosis and progress to ESKD, mainly if not diagnosed and treated promptly.
